# The influence of the fluid nature on femtosecond laser ablation properties of a SiO_2_/Si target and synthesis of ultrafine-grained Si nanoparticles

**DOI:** 10.1039/d0na00317d

**Published:** 2020-06-23

**Authors:** Niusha Lasemi, Christian Rentenberger, Gerhard Liedl, Dominik Eder

**Affiliations:** Institute of Materials Chemistry, Vienna University of Technology 1060 Vienna Austria niusha.lasemi@tuwien.ac.at; Physics of Nanostructured Materials, Faculty of Physics, University of Vienna 1090 Vienna Austria; Institute for Production Engineering and Laser Technology, Vienna University of Technology 1060 Vienna Austria

## Abstract

Nanocrystalline silicon nanoparticles with a median crystallite size of 3–4 nm and several crystalline phases and defects (*e.g.* twin boundary) were produced by femtosecond laser processing of a SiO_2_/Si target in various organic fluids. Furthermore, a nanoscaled amorphous oxide layer and a few atomic layers of a graphite shell were detected in ethanol and 2-butanol correspondingly. The ultrafast laser pulses may manipulate nanostructures at the atomic level and generate a high density of defects; this may be correlated with significant thermal stresses on nanoparticles and rapid condensation of primary nanoparticles with high cooling rates. Size distribution width and a polydispersity index slightly increased with increasing laser fluence in ethanol. In 2-butanol, the maximum ablation volume was observed. The specific ablation rates in 2-butanol and ethanol were approximately five times higher than *n*-hexane. The lowest ablation efficiency in *n*-hexane can be associated with femtosecond laser-induced photolysis and pyrolysis of solvent molecules, as total energy deposition on the material may be reduced due to the formation of carbonaceous products. The roughened zones (average roughness of ∼400 nm) in circumferences of the ablated craters in 2-butanol may be related to a correlation between the erosive power of the vapour bubble collapse and higher pressure at the bubble wall in relatively high dynamic viscosity fluids. Furthermore, sputtering of a pristine surface by releasing nanoparticles from the collective collapse of up-flow vapour bubbles can also contribute to the generation of roughened regions.

## Abstract 2

Femtosecond laser assisted formation of ultrafine-grained Si NPs with a high density of defects. This can correlate with significant thermal stresses on primary NPs, fast cooling of ejected liquid droplets and incomplete ripening processes.

## Graphical abstract

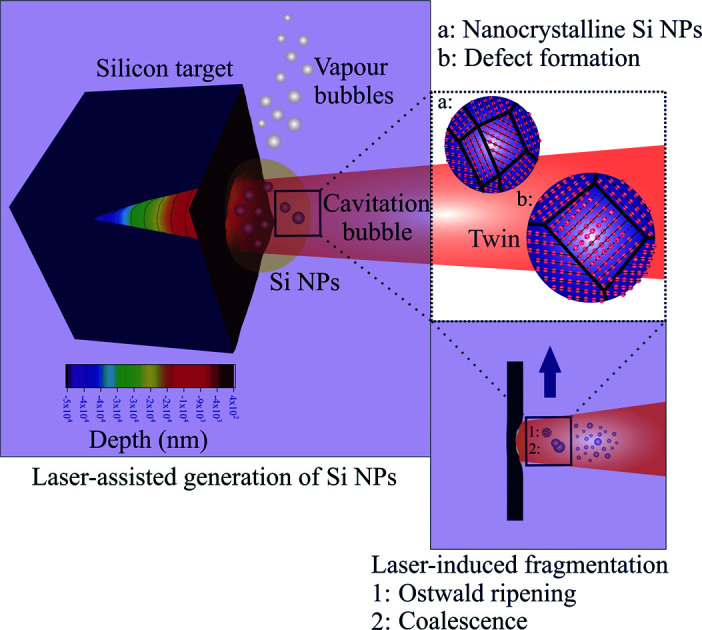

## Introduction

Ultrashort laser pulses assist nanomaterial production *via* nonlinear processes that are broadly present in fundamental and practical studies.^1–8^ High-intensity ultrafast laser pulses at pulse repetition rates (*f*_rep_ ≤ 200 kHz) due to a negligible heat affected zone (HAZ) and low energy losses can establish localized material processing, higher ablation yield and better ablation efficiency.^9–11^ Laser ablation in liquids may serve as a contamination-free method to generate highly pure nanostructures for different scientific and industrial aspects such as electronics, biomedicine, energy conversion and catalysis.^12–17^ Laser ablation in liquids initiates the optical breakdown phenomenon as soon as the magnitude of conduction band electron density in a transparent dielectric medium reaches its critical values of 10^18^ to 10^20^ cm^−3^.^18^ Optical breakdown in fluids induces the oscillations of spherical bubbles; thus, their rebound and collapse processes generate pressure ∼ 10^4^ MPa at the liquid/solid interface that leads to high-speed shockwave diffusion and nonlinear acoustic energy dissipation.^19–21^ High-intensity femtosecond laser pulses trigger electronic excitation due to a larger electric field (∼10^9^ to 10^12^ V m^−1^) compared to the intra-atomic Coulomb field (∼10^9^ V m^−1^) that binds electrons to atoms.^10^ Furthermore, the electronic structure of the material can be perturbed by the interaction of ultrashort pulses with atoms and the creation of a non-equilibrium condition between electrons and atoms.^10–22^ Since the electronic perturbation is substantially faster than the electron-phonon collision time (∼1 ps), the incidence of a breakdown in crystal stability plays a major role in the disintegration of atomic structures and nanomaterial productions. Indeed, phase explosion in femtosecond laser semiconductor processing (≤100 fs) initiated by the occurrence of Coulomb explosion comes from strong repulsive forces between valence band electrons when they reach their critical density; therefore, disintegration and fragmentation of material takes place due to the destabilization of the covalent bonds of atoms in the crystal structure.^23^

Silicon nanoparticles (Si NPs) have been extensively employed in high capacity lithium-ion battery anodes,^24^ high-quality sensors^25^ and microelectronics.^26^ They are also coupled with graphene to create high-performance hybrid photodetectors.^27^ Si NPs have been widely used in biomedical fields due to their biocompatibility and great specific capacity for therapeutics loading.^28–30^ Additionally, Si NPs due to their biodegradable characteristics can straightforwardly transform to orthosilicic acid Si(OH)_4_ which is naturally excreted through urine from the body.^31,32^ Since, a clean production technique is necessary, laser ablation in liquids can serve as a ligand-free technique to produce highly pure nanostructures. In addition to laser parameters, the effect of the liquid nature on size, crystallinity and final composition of nanomaterials is of importance.^7,33–35^ Up to now, there exist a few types of research studies regarding Si NP generation by laser ablation in liquids at pulse durations from femtoseconds up to nanoseconds.^36–46^ In this paper, nanocrystalline Si NPs with several crystalline phases and defects are produced by using a femtosecond laser ablation technique in ethanol, 2-butanol and *n*-hexane. The effects of fluid composition and laser fluence on size distribution, polydispersity and crystallinity of Si NPs are studied. Furthermore, the specific ablation rate of the femtosecond laser processed SiO_2_/Si target along with 3D (three dimensional) mapping analyses of ablated craters is presented. Finally, hydrodynamic properties of colloidal Si NPs (*e.g.* diffusion and friction coefficients) with respect to solvent dynamic viscosity and Stokes radius are evaluated.

## Experimental

### Materials and methods

Silicon wafers, single side polished (SSP), p-type, boron doped, 1–30 Ω cm, with a diameter of 150 mm, thickness of 0.67 mm and ∼300 nm of silica (SiO_2_) coating on the silicon wafer *via* a dry thermal oxide procedure, were provided from SIEGERT WAFER company. Silicon wafers were cut into 9 × 9 mm^2^ pieces and cleaned ultrasonically using acetone and isopropanol in 10 min to eliminate any chemical contamination. The solvents were ethanol, 2-butanol, and *n*-hexane (Sigma-Aldrich; p.a.).

**Fig. 1:**
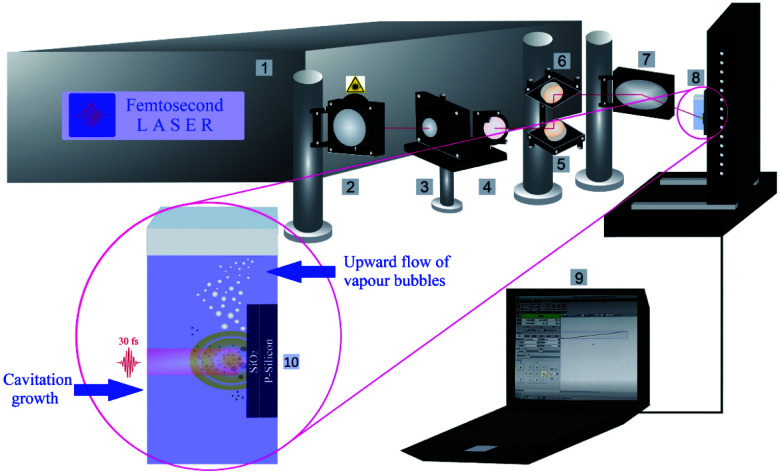
The schematic of the laser ablation set up; 1: femtosecond laser system, 2: mirror, 3: polarizer, 4: beam splitter, 5 & 6: reflecting mirrors, 7: parabolic focusing mirror, 8: target position on motorized XYZ-scanning stage, 9: CNC controller program and 10: nanoparticle synthesis procedure (cavitation bubble expansion along with the formation of up-flow vapour bubbles).

Femtosecond laser processing of SiO_2_/Si targets in liquids was done by using a femtosecond titanium–sapphire (Ti:sapphire) laser at a wavelength of 800 nm, maximum power of ≤1 W, pulse duration of 30 fs and repetition rate of 1 kHz. To have better amplification, the femtosecond laser system consisted of a commercial femtosecond system as a seeding laser (SPECTRA-PHYSICS®, 800 nm, ≤400 mW, 10 fs, 75 MHz), and an Nd:YAG pumping laser emitting at 532 nm. The femtosecond laser set up has been fully described in the previous work.^7^ The schematic of the experimental set up is shown in Fig. 1.

A powermeter (OPHIR Photonics) was positioned after a variable attenuator to control the intensity ratio of the output beam. A variable attenuator consists of a beam splitter and a Brewster (thin film) polarizer including a quartz half waveplate. The focus position in various liquid media was experimentally evaluated by microscopically measuring the ablated areas on a silicon target as a function of the distance of a parabolic mirror (focal length 101.6 mm). Laser ablation in liquids was performed inside precision cells (Hellma Analytics) made of quartz glass with a thickness of 10 mm. A horizontal beam was established to hinder any optical instability (*e.g.* defocusing due to vapour bubble accumulation) during laser ablation in liquids. A femtosecond laser system is equipped with a motorized XYZ-scanning stage that was controlled by a bCNC program. A Gaussian beam distribution is anticipated; therefore, the diameter of the ablated area (*D*) is estimated by optical microscopy (Leica Microsystems CMS GmbH) and is associated with a Gaussian beam radius (*w*_0_). Pulse energy (*E*) and laser fluence (*F*) are correlated by;^47^
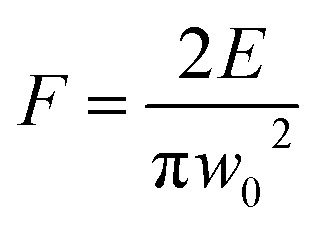


Ultrashort pulses (≤100 fs) broaden as they travel through the liquid media.^48^ Thus, the final pulse durations Δ*t*_out_ for various organic fluids (Table 1) go beyond 30 fs while they remain in the femtosecond range.

**Table 1:** Summary of experimental parameters and fitting results (@800 nm, 1 kHz, *N* = 1000, 30 fs)

Sample	Solvents	*F*/J cm^−2^	*E*/μJ	Δ*t*_out_/fs	*x*_c_/nm	IQR
a	Ethanol	2.94	150	49	9.58 ± 0.19	0.82
b	Ethanol	4.90	250	49	11.17 ± 0.24	1.02
c	Ethanol	6.86	350	49	12.32 ± 0.16	0.65
d	Ethanol	8.82	450	49	13.06 ± 0.36	1.48
e	Ethanol	10.78	550	49	14.11 ± 0.21	0.85
f	2-Butanol	4.42	250	82	8.86 ± 0.17	0.71
g	*n*-Hexane	10.46	250	64	6.97 ± 0.51	2.11

### Profilometry

A DektakXT stylus profiler equipped with a Bruker's intuitive Vision64™ user interface and a Bruker 3D optical microscope system was used to measure the ablation depth, cavity volume and average roughness along with 3D map scanning analyses. A stylus force of 3 mg was applied at the surface of silicon wafers; the radius of the stylus was 2 μm. To have better results in map scan measurements, a map resolution of 10 μm per trace was applied.

### Characterization techniques (HRTEM, TEM, SAED, and EDX)

Silicon nanoparticles were characterized by applying various transmission electron microscopy (TEM) techniques. Bright field (BF-TEM) and dark field (DF-TEM) images as well as selected area electron diffraction (SAED) patterns were taken by using a Philips CM200 TEM (LaB6 cathode, acceleration voltage of 200 kV) equipped with a Gatan Orius CCD camera. The crystallographic data analyses and phase identification of Si NPs were carried out by comparing the collected data with those in the AtomWork database.^49^ Feret size distribution studies were done by evaluation of seven TEM frames (700 × 700 nm) using Gatan microscopy software. Chemical composition analyses were performed by energy-dispersive X-ray spectroscopy (EDX) in connection with TEM. In addition, high resolution transmission electron microscopy (HRTEM) was performed with the FEI Titan 80–300 equipped with an imaging Cs-corrector. Sample preparation for TEM characterization experiments was done by placing several droplets of the colloidal dispersion (after 5 min ultrasonic irradiation) on a lacey carbon film (copper grid) followed by drying in air at room temperature.

### Dynamic light scattering (DLS)

DLS analyses were performed by using a ALV/CGS-3 compact goniometer system that contains a helium-neon laser as a light source (@632.8 nm, power of 22 mW). To avoid contaminations, solvents were filtered by using 0.45 μm polypropylene filter (VWR). Colloidal Si NPs were placed under 5 min ultrasonic irradiation before performing DLS experiments. All experiments were done at room temperature and a goniometer angle of 90°. Stokes radius measurements were carried out by analyses of correlation-function followed by an intensity size distribution (unweighted radius).

## Results and discussion

### Surface profile analyses of the ablated target

**Fig. 2:**
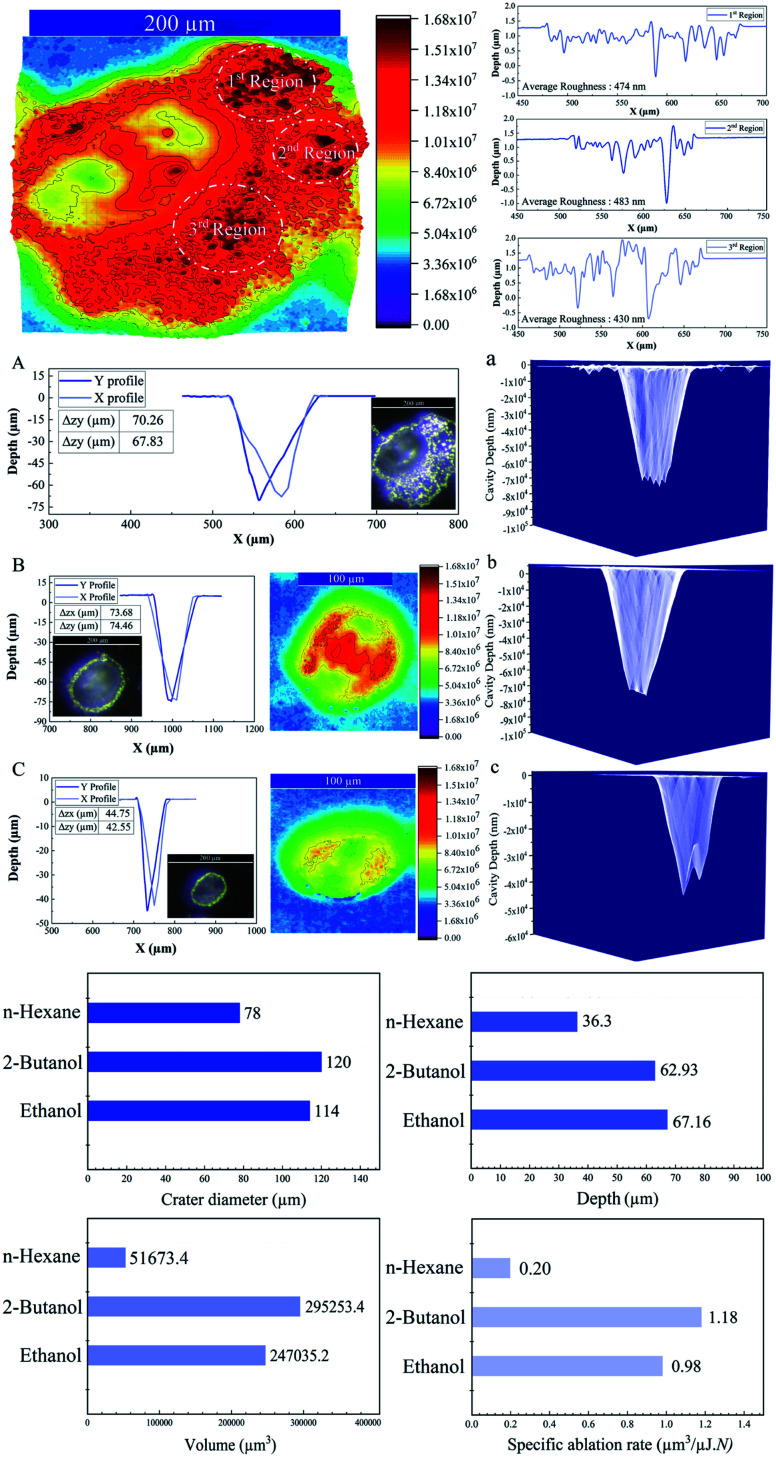
Upper image is a 3D reconstructed optical image of an ablated crater with measured average roughnesses in 2-butanol. Depth profile analyses of femtosecond laser drilled cavities on the SiO_2_/Si target along with optical images, 3D reconstructed images and 3D mapping plots (*N* = 1000; *E* = 250 μJ, 1 kHz) are shown; A & a: in 2-butanol B & b: in ethanol C & c: in *n*-hexane.

The 3D depth profile analyses, surface roughness measurements and 3D reconstruction from optical microscopic images of craters of femtosecond laser processed SiO_2_/Si targets at *E* = 250 μJ and pulse number (*N* = 1000) are presented in Fig. 2.

Femtosecond laser induced filamentation^4^ causes a decrease in ablation efficiency. Since, femtosecond laser ablation of Ag and Au targets in liquids presented a maximum nanoparticle productivity at pulse energies around 200 μJ;^4,50^ hence, the energy around 250 μJ is considered to reduce its contribution in energy losses and its critical effect on cavity depth, width and volume.

A conical shape of ablation depth was observed that is correlated with the Gaussian beam profile with the highest intensity in the center. Therefore, to simplify the estimation, the volume of a cone is evaluated to calculate the ablation volume per cavity. The volume of the cone can be written as; 
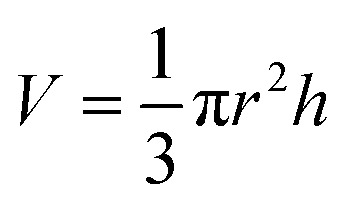
 where *V* is the net missing volume of the crater, and *r* and *h* are the crater radius and depth correspondingly. To be precise, the average ablation depths and volumes in each fluid are calculated by studying several ablated craters. The data show that the estimated specific ablation rate^51^ in 2-butanol is higher than ethanol and *n*-hexane (*cf.*Fig. 2). In water and glycols, persistent and long-lasting bubbles with life times from microseconds to milliseconds were observed in the literature^52,53^ that critically affect the ablation efficiency. However, as the specific ablation rate in 2-butanol with relatively high dynamic viscosity is higher than ethanol and *n*-hexane, it is concluded that the formation of persistent bubbles in 2-butanol can be neglected. On the other hand, previous research showed that the increase of incubation behaviour of nickel in butanol was correlated with solvent decomposition and deposition of the carbon film in the crater.^54,55^ These processes can increase laser absorptivity and subsequently, the ablated volume. In the present study, the optical microscopy results revealed the creation of a roughened area at the circumference of the craters in 2-butanol in contrast to ethanol and *n*-hexane. To create a better visualization, 3D reconstructed profiles of the optical images from ablated craters are added. The total average surface roughness was measured by Vision64 Map™ software *via* studying the roughened parts around several ablated areas region by region. The measured average roughness in 2-butanol is estimated to be ∼400 nm. The lowest and highest average roughness was ∼200 to ∼700 nm, respectively.

In fact, the localized thermalization of liquids (*e.g.* focal position) by using ultrashort laser pulses can trigger fluid phase transition and induce the formation of optic cavitation (*e.g.* vapour bubbles). The mathematical simulation disclosed the correlation between the vapour pressure at the bubble wall concerning surface tension and dynamic viscosity;^56^ thus, the mechanical impact resulting from the erosive power of vapour bubble collapses can be higher in 2-butanol than ethanol and *n*-hexane.

In addition, the as-released Si NPs after a final collapse of cavitation bubble can be trapped by vapour bubbles; therefore, their collapses can divert the particles in the direction of the target position either in the cavity or pristine area simultaneously. Thus, abrasive power resulting from the physical sputtering phenomenon due to the bombardment of the surface of the material with already released particles can be a further reason to generate visible rough zones on the surface of the SiO_2_/Si substrate around the craters. Also, optical microscopy reveals that these roughened zones are accumulated surrounding the half of the ablated area (*cf.*Fig. 2A); this can be related to horizontal beam delivery that provides vertical up-flow of vapour bubbles (*cf.*Fig. 1) and subsequently to the collective collapse of oscillating vapour bubbles in one direction. Furthermore, the bubble wall may act as a focusing lens as a result of a partial scattering of laser light by already formed nano/microbubbles; this may also contribute to the formation of roughened regions.

The lowest specific ablation rate was noticed in *n*-hexane similar to toluene;^51^*n*-hexane and toluene are both non-polar solvents with a higher number of carbon contents. Therefore, the highest amount of laser energy can be employed for pyrolysis and photolysis of solvent molecules and form carbonaceous products.^33,34^ These carbon products and graphite flakes can sufficiently absorb incoming laser pulses and reduce energy deposition on the substrate. In addition, lower dynamic viscosity of *n*-hexane with respect to 2-butanol and ethanol can affect fluid flow velocity; thus, a chaotic ablation process is expected. This can affect ablation efficiency, leading to the smallest crater width, in comparison with 2-butanol and ethanol media. 3D mapping analyses of ablated craters (*cf.*Fig. 2a) exhibit a roughened area at the center of the cavities in ethanol and 2-butanol. These findings are in line with the ablation results of metallic craters where micron-sized granular structures were observed.^57^

### Analysis of nanoparticles

**Fig. 3:**
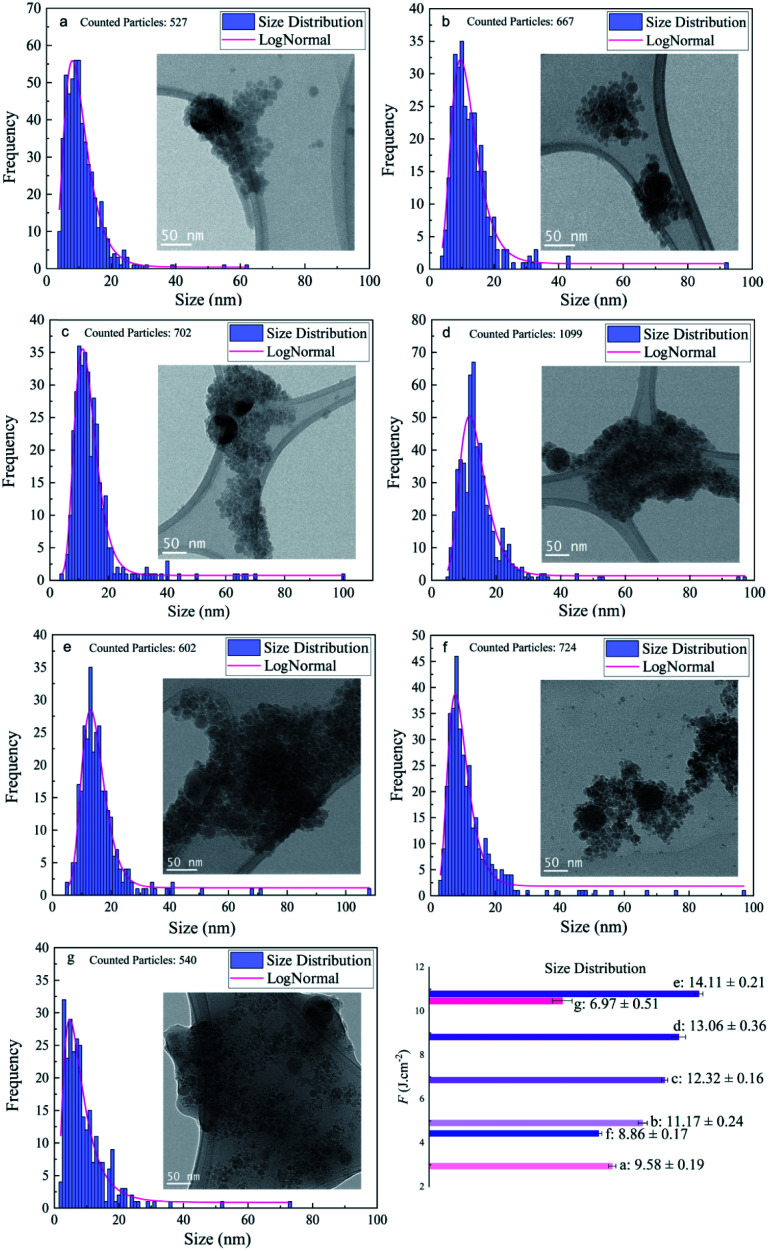
Feret size distribution study of femtosecond laser synthesized silicon nanoparticles (*N* = 1000, 1 kHz); (a) ethanol, *F* = 2.94 J cm^−2^, (b) ethanol, *F* = 4.90 J cm^−2^, (c) ethanol, *F* = 6.86 J cm^−2^, (d) ethanol, *F* = 8.82 J cm^−2^, (e) ethanol, *F* = 10.78 J cm^−2^, (f) 2-butanol, *F* = 4.42 J cm^−2^, and (g) *n*-hexane, *F* = 10.46 J cm^−2^.

Regarding the fluence dependency, the median size of Si NPs in ethanol is slightly increased at higher *F* (Fig. 3 and Table 1). This is in good agreement with theoretical and experimental observations.^1,58,59^

In fact, for longer pulse durations (*e.g.* nanosecond regime) the effect of *F* on the size of colloidal ZnO,^60^ Ni/NiO_*x*_ (ref. 55) and Fe/Fe_*x*_O_*y*_ (ref. 13) nanoparticles showed the same trend. Obviously, the photon interaction with nanoparticles can increase the frequency of particle collisions and growth; thus, higher laser intensity induces higher Brownian motion, greater particle–particle collisions and subsequently larger nanoparticles. Indeed, large nanoparticles can be formed by particle growth due to the coexistence of two ripening mechanisms: coalescence and Ostwald ripening; however, kinetic simulations of crystal growth revealed the priority of the coalescence process that includes crystallographic reorientation and realignment of two attached particles.^61^ The theoretical^58,59^ and experimental^62–65^ studies of cavitation bubble dynamics and laser ablation of metals in confined fluid media exposed the formation of two specific average sizes of nanoparticles. Primary nanoparticles (∼10–20 nm) were produced due to the fast cooling and condensation in a low density mixing region and secondary nanoparticles (∼50–60 nm) were generated by the breakup of the superheated molten metal layer at a plume–fluid interface induced by the Rayleigh–Taylor instabilities.^59^ Furthermore, agglomeration of primary NPs confined inside the cavitation bubble can be responsible for generation of larger nanoparticles.^64,66^ Further particle diffusion and growth by ripening processes occur after the final collapse of the cavitation bubble.^65^

In addition to the median sizes (*x*_c_) for positively skewed lognormal size distributions, the interquartile range (IQR) was measured and is presented in Table 1. IQR values estimate deviations from median sizes.^7^ In ethanol, no real correlation between IQR and different laser fluences is found. However, at the given energy of *E* = 250 μJ, the lowest IQR value is calculated in 2-butanol and can be correlated with the lower polydispersity of Si NPs compared to ethanol and *n*-hexane. Si NPs showed a nearly monomodal size distribution and less IQR values in comparison to metallic nanoparticles; one can suggest a non-thermal interaction of ultrashort laser pulses with the semiconductor target may initiate homogenous phase explosion and uniform lattice disintegration. For a given energy of 250 μJ, the median size of the Si NPs changes inversely with increasing carbon number of the organic solvent. The smallest feret size of Si NPs is observed in *n*-hexane and 2-butanol.

Additionally, analyses of TEM images acquired from particles in *n*-hexane indicated that they were embedded in the carbonaceous matrix. It is assumed that the graphitization of nanoparticles can offer capping agent characteristics; the amorphous carbon can also create steric hindrance to avoid nanoparticles from further aggregation and coalescence.^7,33^ In fact, ultrafast pulse interaction with liquid media induces localized thermalization, nonlinear energy absorption and subsequent photoionization of the solvent.^67,68^ Femtosecond laser pulses can create high-temperature plasma (5000–7000 K);^69^ hence, thermal pyrolysis of solvent molecules occurs due to the formation of a supercritical temperature liquid.^59^ Moreover, a direct photolysis of solvent molecules by high-intensity femtosecond laser pulses is highly probable.^34^ In order to assess crystallinity and phases, all the Si samples in ethanol have been studied by SAED. PASAD-tools were applied to calculate reciprocal lattice distances and obtain crystallographic data.^70^ The measured lattice distances were compared to those in the database.^49^ At all laser fluences, crystalline structures of the Si nanoparticles were observed. Many Si NPs, especially at higher *F*, contained crystalline defects. As femtosecond pulses are much shorter than the electron-lattice relaxation time (10^−10^ to 10^−12^ s),^71,72^ due to the instant cooling of plasma plume and rapid solidification of nanoparticles,^59^ defect formation can be simply instigated.

**Fig. 4:**
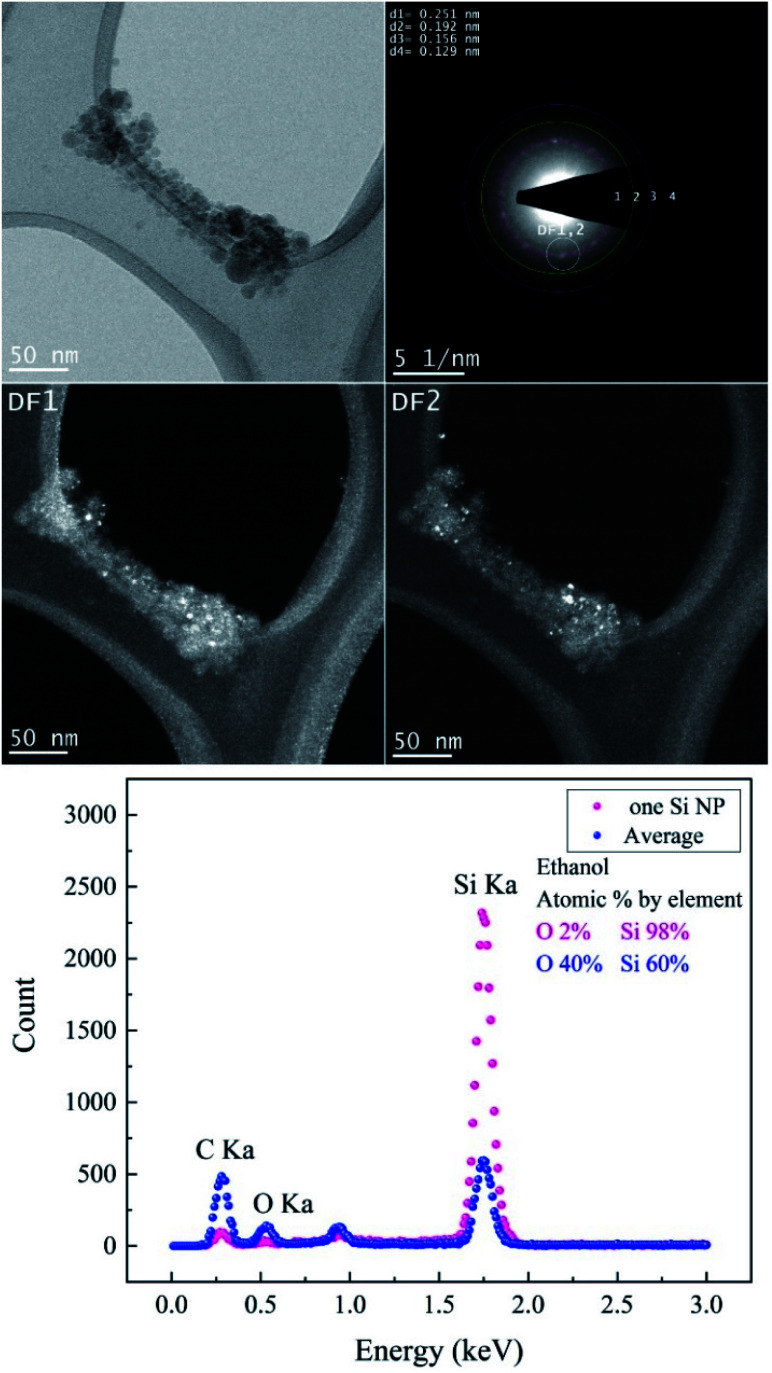
The femtosecond laser-synthesized Si nanoparticles in ethanol. (*N* = 1000, *F* = 4.90 J cm^−2^): BF-TEM image (upper left), SAED pattern (upper right) and DF-TEM images related to different diffracted electron beams along the 1^st^ ring. The EDX spectrum contains an average of several Si NPs and one of a single Si NP.

TEM images of femtosecond laser synthesized Si NPs in ethanol at moderate *F* = 4.90 J cm^−2^ are shown in Fig. 4. Dark-field images are achieved by using objective aperture in the diffraction plane at different positions along the first ring. DF1,2 images are related to the bright spot on the first ring that is correlated with crystalline cubic SiC.

**Fig. 5:**
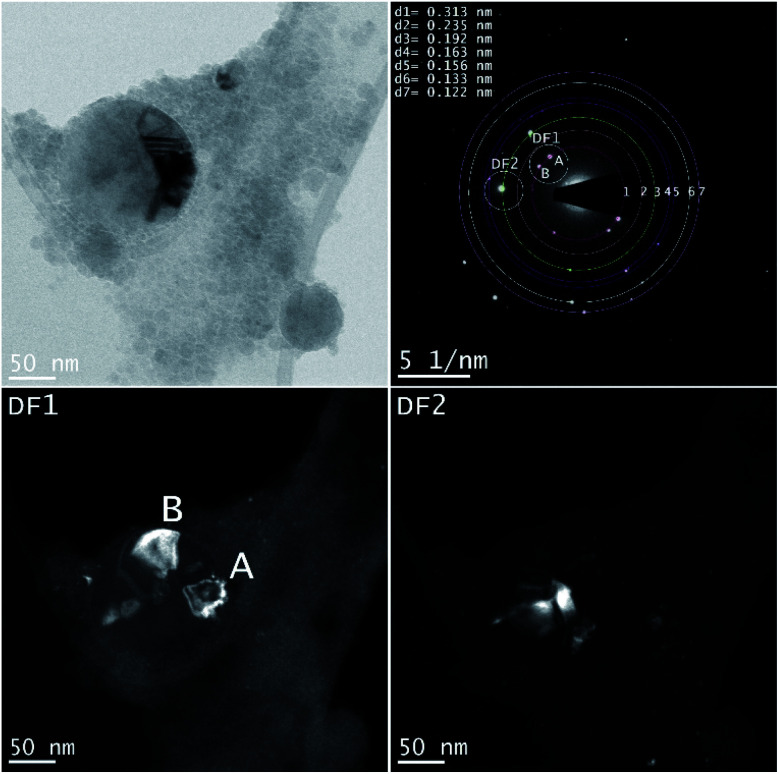
The femtosecond laser-synthesized Si nanoparticles in ethanol. (*N* = 1000, *F* = 10.78 J cm^−2^). The BF-TEM image (upper left), SAED pattern (upper right) and the DF-TEM images related to different diffracted electron beams along the 1^st^ and 3^rd^ ring.

The crystallographic data of the evaluated SAED pattern shown in Table 2 revealed the presence of various phases. The EDX spectrum (*cf.*Fig. 4) from one large particle indicated a high concentration of Si of ∼98% and a low content of oxygen; on the other hand, the oxygen concentration of agglomerated small Si NPs is considerably higher. Therefore, it can be assumed that the particles are covered by a thin oxide shell since many small particles have a larger surface area; a potential carbon content cannot be evaluated due to the carbon film as a substrate. BF-TEM, SAED and DF-TEM of Si NPs in ethanol at the highest *F* are presented in Fig. 5.

**Table 2:** Crystallographic data and phase identifiers of Si NPs in ethanol (*N* = 1000, *F* = 4.90 J cm^−2^)

Material	Crystal system	Lattice distance/nm	Miller indices	Pearson symbol	Space group
SiC	Cubic	0.251	[111]	cF8	*F*4̄3*m*
Si	Cubic	0.192	[220]	cF8	*Fd*3̄*m*
Si	Cubic	0.156	[222]	cF8	*Fd*3̄*m*
Si	Orthorhombic	0.129	[301]	oI4	*Imma*

A larger NP shows a defect structure that may be related to a twin boundary defect that is a special type of a planar defect. Objective aperture at the position of the first ring (indicated in the SAED pattern) is used to form the DF1 image. The bright spots (A, B) in the SAED pattern can be correlated with cubic Si [111] and form the bright areas in the DF1 image. The DF2 image is related to the [220] reflection of cubic Si (Table 3).

**Table 3:** Crystallographic data and phase identifiers of Si NPs in ethanol (*N* = 1000, *F* = 10.78 J cm^−2^)

Material	Crystal system	Lattice distance/nm	Miller indices	Pearson symbol	Space group
Si	Cubic	0.313	[111]	cF8	*Fd*3̄*m*
Si	Tetragonal	0.235	[200]	tI4	*I*4_1_/*amd*
Si	Cubic	0.192	[220]	cF8	*Fd*3̄*m*
Si	Cubic	0.163	[311]	cF8	*Fd*3̄*m*
Si	Cubic	0.156	[222]	cF8	*Fd*3̄*m*
Si	Tetragonal	0.133	[301]	tI4	*I*4_1_/*amd*
Si	Hexagonal	0.122	[110]	hP2	*P*6_3_/*mmc*

**Fig. 6:**
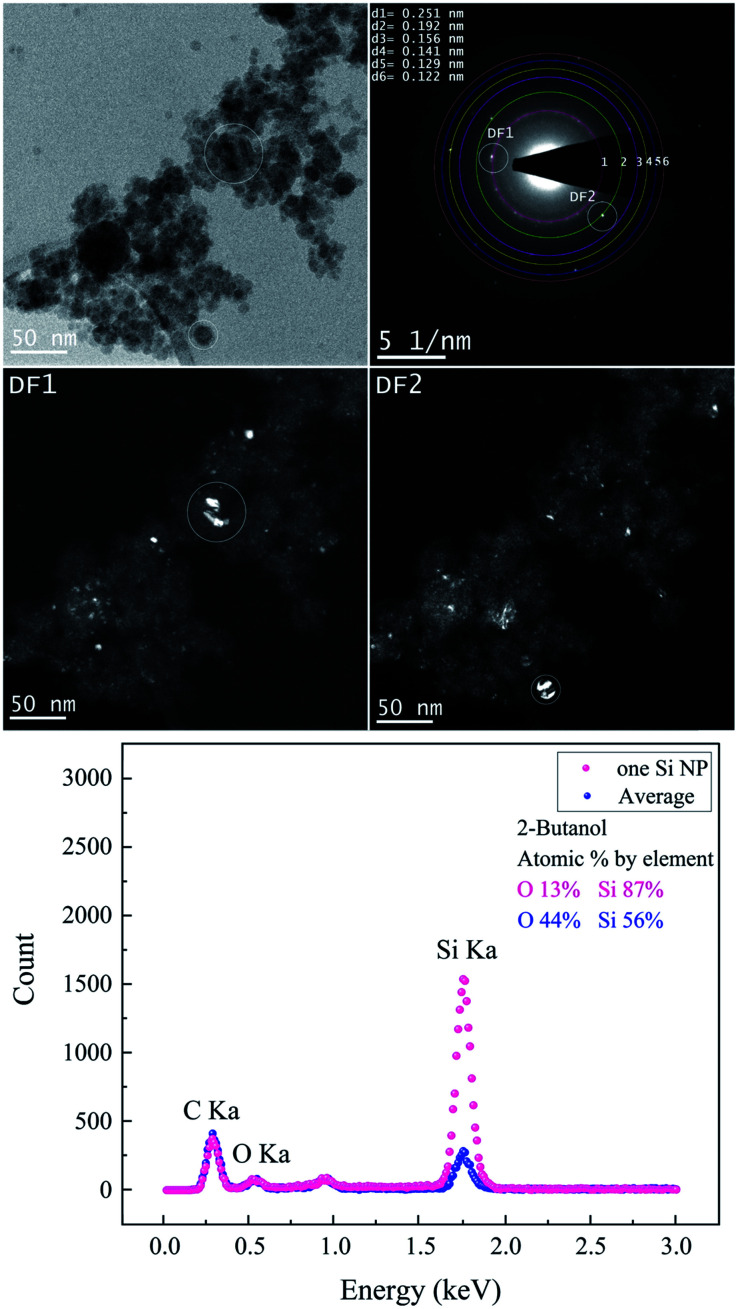
The femtosecond laser-synthesized Si nanoparticles in 2-butanol. (*N* = 1000, *F* = 4.42 J cm^−2^). The BF-TEM image (upper left), SAED pattern (upper right) and the DF-TEM images related to different diffracted electron beams along the 1^st^ and 2^nd^ ring. The EDX spectrum of several Si NPs shown in the BF-TEM and that of one single Si NP.

Both BF-TEM and DF-TEM images, as well as the SAED pattern and EDX spectrum of Si NPs produced in 2-butanol are shown in Fig. 6. Si NPs with various crystal systems are synthesized in 2-butanol (Table 4). Bright areas in the DF1 image (related to bright spot on the first ring with a lattice distance of 0.251 nm) correspond to particles with a cubic SiC structure that is observed analogous to ethanol. In contrast, DF2 image acquired by intensity on the second ring shows bright areas with the cubic Si structure.

**Table 4:** Crystallographic data and phase identifiers of Si NPs in 2-butanol (*N* = 1000, *F* = 4.42 J cm^−2^)

Material	Crystal system	Lattice distance/nm	Miller indices	Pearson symbol	Space group
SiC	Cubic	0.251	[111]	cF8	*F*4̄3*m*
Si	Cubic	0.192	[220]	cF8	*Fd*3̄*m*
Si	Cubic	0.156	[222]	cF8	*Fd*3̄*m*
Si	Cubic	0.141	[332]	cI16	*Ia*3̄
Si	Orthorhombic	0.129	[301]	oI4	*Imma*
Si	Hexagonal	0.122	[110]	hP2	*P*6_3_/*mmc*

**Fig. 7:**
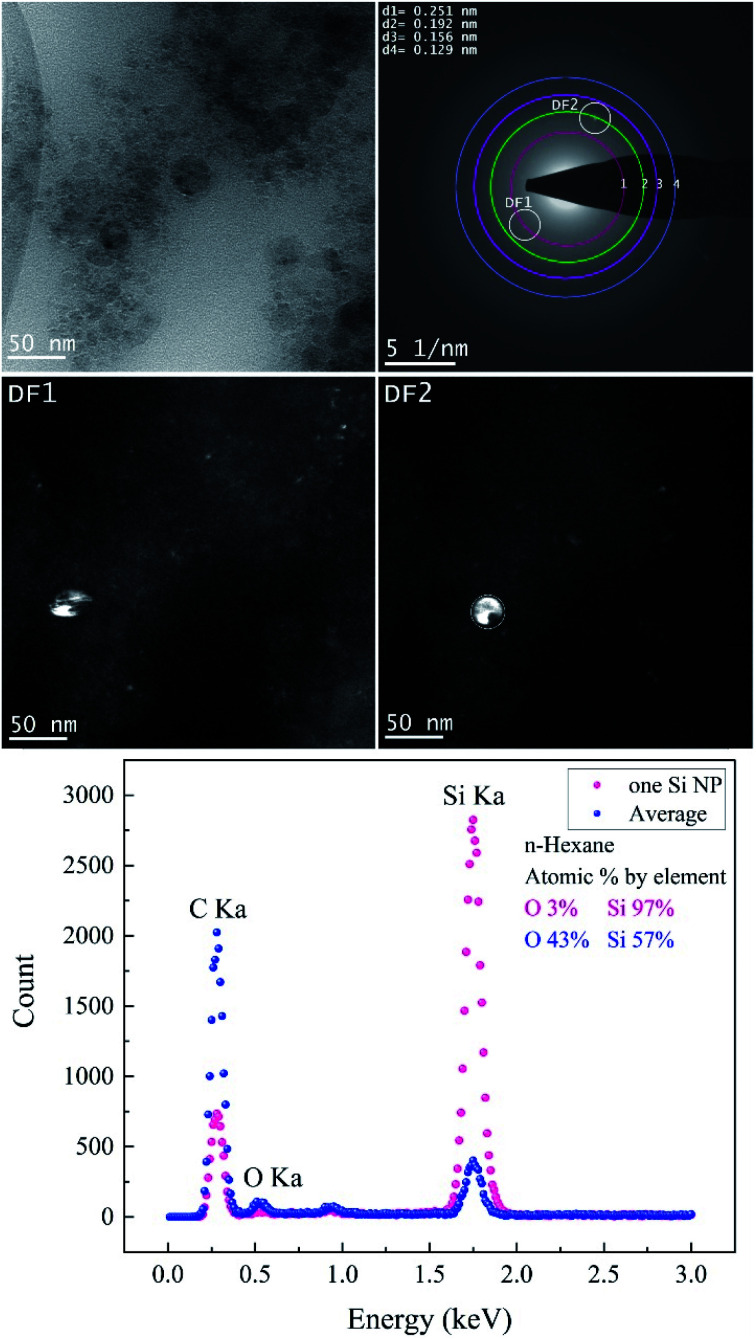
The femtosecond laser-synthesized Si nanoparticles in *n*-hexane. (*N* = 1000, *F* = 10.46 J cm^−2^). The TEM bright-field image, SAED pattern (upper right) and dark-field images related to different diffracted electron beams along the 1^st^ and 2^nd^ ring. The EDX spectrum from several small Si NPs and one Si NP.

Fig. 7 and Table 5 show the results obtained from the analyses of Si NPs in *n*-hexane. BF-TEM images, electron diffraction and EDX measurements indicated that Si NPs are embedded in a thick layer of amorphous carbon; indeed, the probability of pyrolysis and photolysis is higher due to the existence of a higher number of carbon molecules.

**Table 5:** Crystallographic data and phase identifiers of Si NPs in *n*-hexane (*N* = 1000, *F* = 10.46 J cm^−2^)

Material	Crystal system	Lattice distance/nm	Miller indices	Pearson symbol	Space group
SiC	Cubic	0.251	[111]	cF8	*F*4̄3*m*
Si	Cubic	0.192	[220]	cF8	*Fd*3̄*m*
Si	Cubic	0.156	[222]	cF8	*Fd*3̄*m*
Si	Orthorhombic	0.129	[301]	oI4	*Imma*

During the ablation process, laser interaction with higher carbon content solvents such as toluene, *n*-hexane, acetone and butanol may trigger the graphitization of nanoparticles^7,55^ and production of amorphous carbon flakes.^17,33,34,73–75^ This is in good agreement with depth profile analyses results as the lowest specific ablation rate was observed in *n*-hexane, in contrast with ethanol and 2-butanol. Besides carbonaceous products, Si NPs are also surrounded by a carbon matrix; thus, these secondary products can also contribute to the absorption of the incoming laser beam and increase of energy losses. SAED analyses (Table 5) expose the existence of cubic SiC (lattice distance of 0.251 nm) and cubic Si (lattice planes [220]) similar to ethanol and 2-butanol. Particles corresponding to these two structures are seen bright in DF1 and DF2, respectively.

**Fig. 8:**
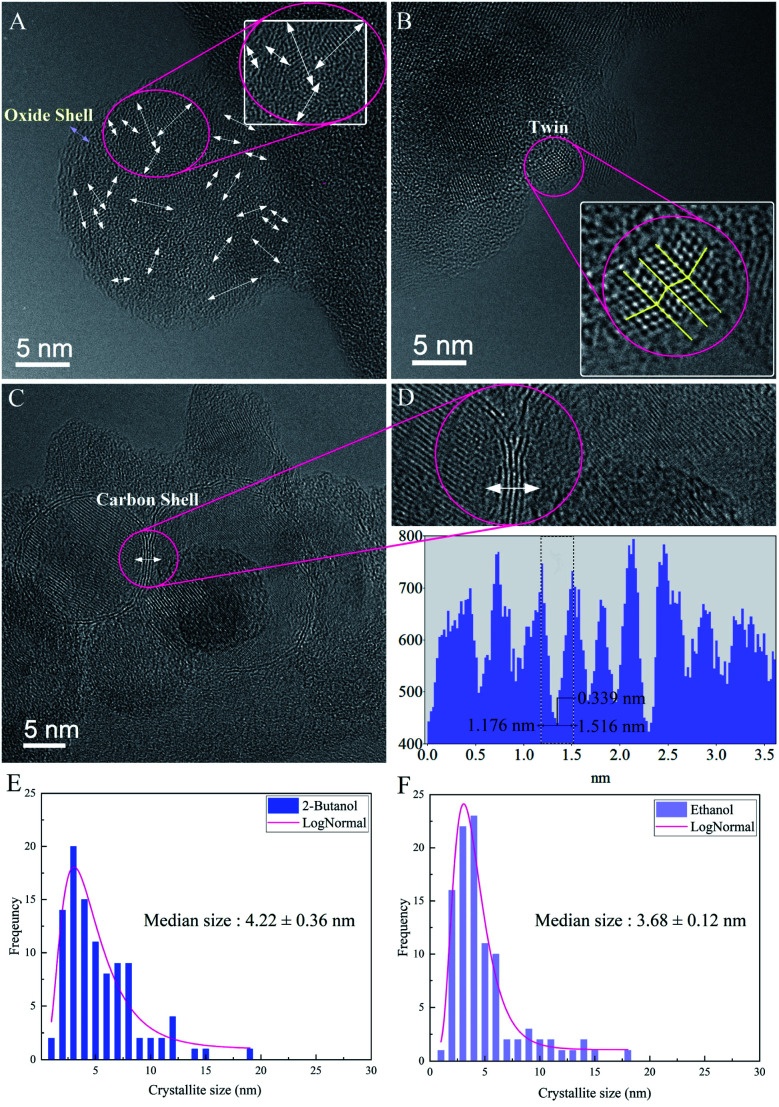
HRTEM images of nanocrystalline Si NPs; (A) atomic orientations of an ultrafine-grained Si NP with an amorphous oxide shell in ethanol (*N* = 1000, *F* = 4.90 J cm^−2^), (B) twin boundary defect in a Si NP in ethanol, (C) Si NP in 2-butanol (*N* = 1000, *F* = 4.42 J cm^−2^) with a few atomic layers of the graphite shell, (D) zoom plot of graphite fringes with respective HRTEM profiles, (E) study of the average crystallite size of Si NPs in ethanol, (F) study of the average crystallite size of Si NPs in 2-butanol.

High-resolution electron microscopy confirmed the formation of crystalline Si NPs and revealed that nanoparticles are nanostructured with a high density of grain boundaries and crystallites with a median size of ∼3 to ∼4 nm in ethanol and 2-butanol, respectively (*cf.*Fig. 8). In order to form NPs with an internal nanoscaled structure, ripening and solidification processes can be considered. In the case of ripening, attached primary particles of nanometer size coalesce but crystallographic reorientation and realignment of attached particles are incomplete and an internal structure with a high density of defects is formed. On the other hand, nanostructured NPs can also be formed during fast solidification of ejected liquid droplets if the cooling rate for solidification is high. Experimental and theoretical studies revealed that during laser ablation in liquids the cooling rates in the order of 10^10^ K s^−1^ and 10^12^ K s^−1^ are expected respectively.^59,76^

In ethanol, nanoscaled amorphous oxide shells were observed in agreement with EDX analyses; it may be correlated with the formation of a native oxide layer during cooling and solidification phases. However, HRTEM in 2-butanol revealed contrast fringes around Si NPs with distances of ∼0.33 nm that can be related to lattice planes of graphite whose formation is due to solvent decompositions by high-intensity laser beams. This is similar to previous HRTEM results of femtosecond laser-assisted graphitization of Ni/Au bimetallic oxide NPs in butanol.^7^

Furthermore, in several nanocrystalline Si NPs produced in ethanol and 2-butanol, twins were atomically resolved (*cf.*Fig. 8). Generally, twins can be formed either during solidification and growth (growth twins) or by deformation of the solid according to coordinated atomic displacements (deformation twins). In the present case of laser ablation, both twin formation processes can take place since growth under non-equilibrium conditions or thermal stresses on particles that are expected to occur, favor twin formation.^77,78^ One can assume that by increasing *F* the rate of thermal stresses on crystalline structures increases and nanoparticles with different phases containing various crystallographic defects can be formed as a result of formation of a supercritical temperature liquid, high pressure–temperature cavitation bubble and post-irradiation of already synthesized nanoparticles.

**Fig. 9:**
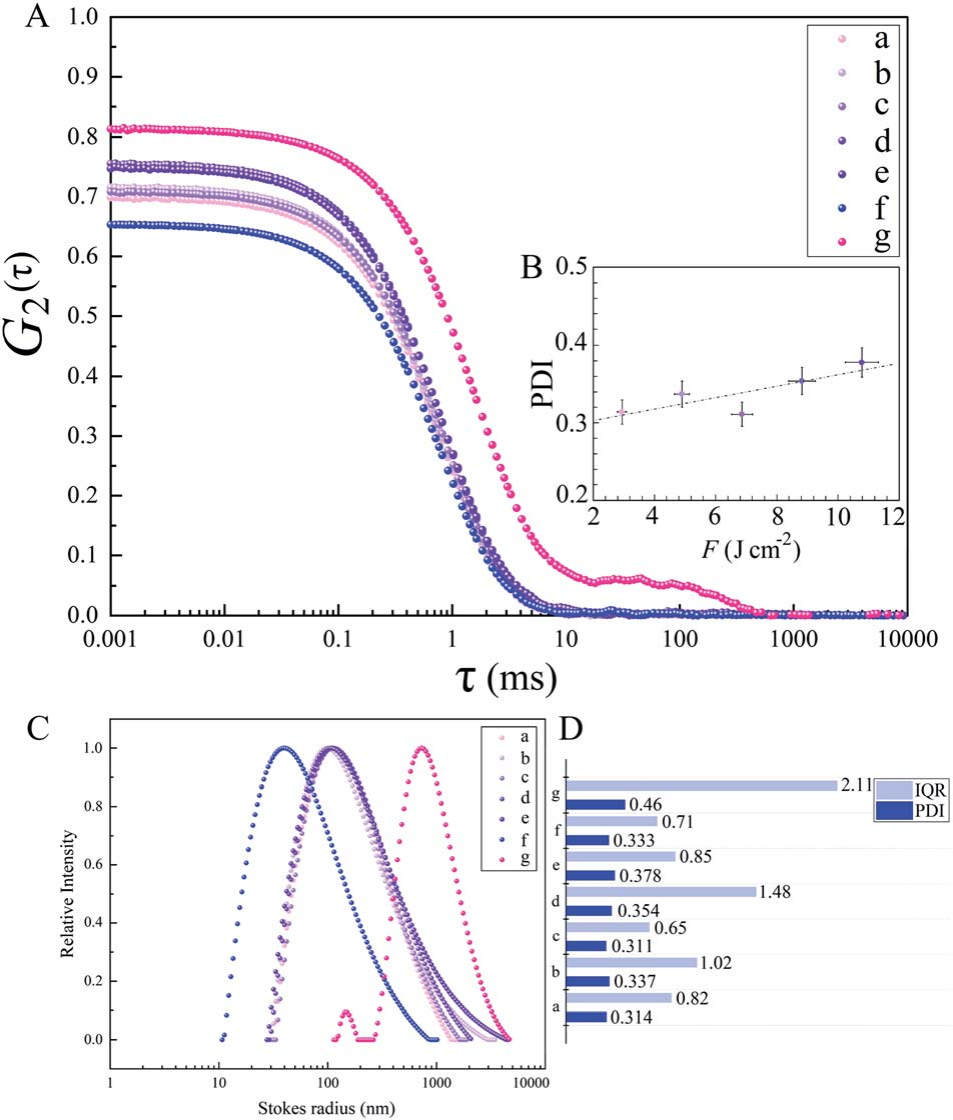
Dynamic light scattering of Si nanoparticles in organic solvents; (A) autocorrelation function curves *versus* delay time; (B) PDI *versus F* for Si NPs in ethanol; (C) relative scattered intensities *versus* Stokes radius for Si NPs in various organic fluids; (D) comparison of PDI with IQR values; a, b, c, d and e: Si NPs in ethanol; f: Si NPs in 2-butanol; g: Si NPs in *n*-hexane.

The diffusion velocity of femtosecond laser synthesized Si NPs in various organic solvents is measured *via* using DLS. The correlation function *G*_2_(*τ*) curves of the normalized intensity *versus* delay time (*τ*) showed a smooth exponential decay of silicon nanoparticle fluctuations in ethanol and 2-butanol, in contrast with the *n*-hexane solvent (*cf.*Fig. 9). In *n*-hexane, a second decay can be related to carbonaceous species (*e.g.* graphite and amorphous carbon flakes) that can contribute to dynamic light scattering results. The slower rate of decay can be detected for larger Si NPs as they diffuse slower in the liquid media. The translational and rotational diffusion of colloidal silicon nanoparticles in a simple continuous model can be explained by Stokes–Einstein (SE) and Stokes–Einstein–Debye (SED) relations in a hydrodynamic framework respectively^79,80^ that can be described as;
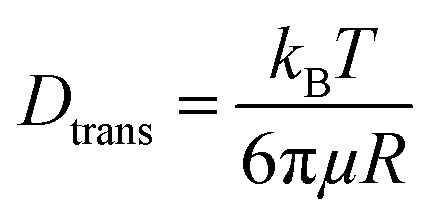
&
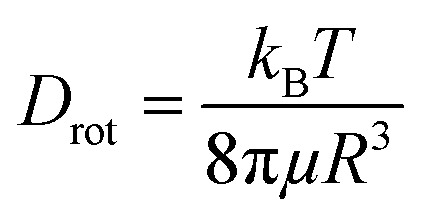
*D*_trans_ is the translational diffusion coefficient; *D*_rot_ is the rotational diffusion coefficient; *R* is Stokes radius; μ is dynamic viscosity; *T* is the absolute temperature and *k*_B_ is Boltzmann constant.

Frictional coefficients of spherical particles can be measured as *f*_trans_ = 6π*μR* and *f*_rot_ = 8π*μR*^3^ and are related to translational and rotational diffusion, correspondingly. Hydrodynamic properties of colloidal Si NPs including Stokes radius, diffusion and friction coefficients are summarized in Table 6. In fact, the fluctuation velocity of colloidal Si NPs can be affected by frictional resistances from the surrounding viscous fluids. However, during laser ablation, the maximum number of NPs is confined inside the cavitation bubbles; thus, the energy of shockwave emissions coming from bubble collapses can affect their actual diffusion and dispersion. In higher dynamic viscosity fluids, frictional forces play a significant role in bubble dynamics (*e.g.* shape). Indeed, bubbles in viscous fluids displayed two distinguished geometrical parts; a spherical bubble cap and a non-spherical specific interlayer that are separated by a distinct rim.^81^ Additionally, cavitation bubble formation in fluids containing nanoparticles may induce a snow-plough effect on synthesized NPs at the gas/liquid interface.^82^

**Table 6:** Hydrodynamic properties of colloidal Si NPs

Sample	*F* (J cm^−2^)	*R* (nm)	*D*_trans_ (μm^2^ s^−1^)	*D*_rot_ (s^−1^)	*f*_trans_ (kg s^−1^)	*f*_rot_ (kg m^2^ s^−1^)
a	2.94	150	2.48	41.4	3.04 × 10^−9^	0.91 × 10^−22^
b	4.90	155	2.40	37.5	3.14 × 10^−9^	1.00 × 10^−22^
c	6.86	157	2.37	36.1	3.18 × 10^−9^	1.04 × 10^−22^
d	8.82	160	2.33	34.1	3.24 × 10^−9^	1.11 × 10^−22^
e	10.78	170	2.19	28.4	3.44 × 10^−9^	1.33 × 10^−22^
f	4.42	59	1.80	194	4.18 × 10^−9^	0.19 × 10^−22^
g	10.46	150	9.20	153	0.82 × 10^−9^	0.24 × 10^−22^

Presumably, the collapse of deformed bubbles in relatively high dynamic viscosity fluids with the influence of hydrodynamic forces at a bubble/liquid interface can all affect the dynamics of Si NP diffusion in fluids.

Dynamic viscosity of 2-butanol (∼3.10 mPa s) is relatively higher than ethanol (∼1.03 mPa s) and *n*-hexane (∼0.29 mPa s); in *n*-hexane, with the lowest dynamic viscosity and subsequently higher Reynolds number, a fast nanoparticle diffusion is expected that leads statistically to an increase of particle collisions and results in the formation of larger nanoparticles. In contrast, the analyses by TEM exhibit Si NPs with the smallest particle size. This can be related to the observed carbonaceous matrix that prevents particles from aggregation. In 2-butanol with the highest dynamic viscosity, the lowest translational diffusion is measured; besides a better colloidal stability due to the graphitization of nanoparticles, the low velocity of nanoparticle diffusion causes less collisions and lower aggregation. The polydispersity index (PDI) resulting from the cumulant fit of intensity correlation curves exhibits a slight increase with increasing *F* in ethanol (*cf.*Fig. 9). Indeed, the degree of electrophoretic velocities of colloidal gold nanoparticles showed a linear increase with respect to increasing laser energies;^5^ clearly, nanoparticle mobility rises at higher laser energies; this can trigger a higher motion, anisotropic diffusion and subsequently orthokinetic aggregation of ablated particles.

Regarding polydispersity, the different trend of IQR values obtained from TEM results is related to statistics, as with DLS, the large number of colloidal nanoparticles can be detected whereas in TEM only a small number of synthesized nanoparticles is observable. As rotational diffusion is directly related to the volume of nanoparticles, a faster rotational diffusion is detected for smaller particles; therefore, Si NPs in 2-butanol with the lowest Stokes radius are shown to have the highest rotational diffusion coefficient and subsequently the lowest frictional forces, in contrast with ethanol and *n*-hexane (*cf.*Table 6). It should be considered that for the classical hydrodynamic diffusion model, constant viscosity is assumed due to the constant temperature, but obviously at elevated temperatures the diffusion can be considerably increased.

## Conclusions

Femtosecond laser ablation of a SiO_2_/Si target in organic fluids resulted in the generation of nanostructured Si nanoparticles. The analyses of Si nanoparticles produced in various liquids showed nearly monomodal lognormal distributions and revealed the formation of different crystal phases (*e.g.* cubic, hexagonal). In ethanol, a relative increase of the nanoparticle size and polydispersity index were detected with an increase of laser fluences; this can be related to increased Brownian motions, particle–particle collisions, anisotropic diffusions and consequently orthokinetic aggregation. The smallest feret diameter was observed in *n*-hexane since the existence of amorphous carbon flakes can induce steric hindrance for collisions of colloidal nanoparticles. In 2-butanol, the small size can be related to the formation of thin graphite shells around Si NPs that acted as a capping agent and slowed down further diffusion and growth. The existence of a graphitic shell around nanoparticles in 2-butanol could be confirmed by high resolution TEM. On the other hand, nanoparticles in ethanol are covered by a native oxide shell. HRTEM also disclosed the defective internal nanostructure of the Si nanoparticles, *e.g.* nanoscaled crystallites (∼3–4 nm in size), and grain and twin boundaries. The occurrence of nanoparticles with small crystallites is compatible with an incomplete coalescence process of primary particles and fast solidification of liquid droplets; therefore, twin formation can be proceeded by both growth and deformation processes. Moreover, the frequency of crystalline imperfections increased in Si NPs at higher fluences in ethanol; this may be correlated with exerted higher thermal stresses on nanostructures due to nucleation in confined plasma, growth in high temperature–pressure bubbles, fast cooling and resolidification of primary nanoparticles and the post-irradiation of already synthesized nanoparticles.

The highest specific ablation rate was observed in 2-butanol; it is assumed the increased absorptivity due to carbon deposition in the crater can be responsible for the higher ablation rate. Furthermore, roughened regions with an average roughness of ∼400 nm in the circumference of ablated craters in 2-butanol were detected. This can suggest a complex mechanism of laser ablation in fluids that includes a series of phases extended over many orders of magnitude in time. In this regard, two proposed mechanisms were considered concerning the generation of roughened regions. Firstly, the mechanical impact coming from the concurrent collapses of up-flow vapour bubbles with a higher pressure at the bubble wall in moderately high dynamic viscosity fluids. Secondly, the erosive power resulting from a physical sputtering by particle bombardment of a pristine surface of the material due to the collective collapse of high pressure-vapour bubbles as they contain ablated materials.

Frictional resistances coming from the surrounding viscous fluids can affect the fluctuation velocity of colloidal Si NPs. In relatively high dynamic viscosity fluids, due to the presence of hydrodynamic forces at the bubble/liquid interface, bubble dynamics (*e.g.* evolution, expansion and collapse) can be different. The lowest nanoparticle productivity was observed in *n*-hexane; this can be correlated with secondary processes that affect ablation efficiency. Laser-induced photolysis and pyrolysis of the liquid media can trigger the formation of carbonaceous compounds (*e.g.* flakes) in *n*-hexane; the intensity correlation curve in *n*-hexane showed a complex exponential decay at a longer delay time that can be related to sedimentation of carbon by-products during DLS measurements. Additionally, the presence of thick layers of carbon matrices embedding Si nanoparticles (as demonstrated by TEM) contributed to higher energy losses within the liquid medium and decreased the ablation efficiency.

## Conflicts of interest

There are no conflicts to declare.

## Supplementary Material
